# Interleukin-1β regulates the expression of glucocorticoid receptor isoforms in nasal polyps in vitro via p38 MAPK and JNK signal transduction pathways

**DOI:** 10.1186/s12950-014-0046-z

**Published:** 2015-01-20

**Authors:** Zhenlin Wang, Peng Li, Qiuhang Zhang, Haili Lv, Junqi Liu, Jinyuan Si

**Affiliations:** Department of Otolaryngology-Head and Neck Surgery, Xuan Wu Hospital, Capital Medical University, 45 Changchun Street, Xicheng District, Beijing, 100053 PR China; Department of Otolaryngology-Head and Neck Surgery, The Third Affiliated Hospital of Sun Yat-sen University, 600 Tianhe Street, Tianhe District, Guangzhou, 510630 PR China

**Keywords:** Chronic rhinosinusitis (CRS), Nasal polyp (NP), Signal transduction, Mitogen-activated protein kinase (MAPK)

## Abstract

**Background:**

To explore the upstream signal transduction mechanisms responsible for the imbalanced expression of glucocorticoid receptor (GR) isoforms in chronic rhinosinusitis (CRS) mucosa.

**Methods:**

An in vitro model of Glucocorticoid resistance was established by inducing nasal polyp tissue with IL-1β. Changes in the protein and mRNA expression of GRα, GRβ and the key enzymes in the p38 MAPK and JNK signal pathways were measured, respectively, before and after being induced with different doses of IL-1β and specific inhibitors of p38 MAPK, JNK, ERK, PI3K and PKC. The Glucocorticoid sensitivity was measured using in vitro Glucocorticoid binding assay. Analysis of variance (ANOVA) was used to analyze the data.

**Results:**

The mRNA and protein expression levels of GRα, GRβ and key enzymes of the p38 MAPK and JNK pathways increased both in time- and concentration-dependent manners in IL-1β-induced nasal polyp tissue. The expression of GRβ increased more significantly than that of GRα, and the GRα/GRβ ratio decreased in time- and concentration-dependent manners. Statistically significant differences were found in the GRα/GRβ ratio and the mRNA expression of phospho-p38 MAPK and phospho-JNK between the IL-1β-induced groups and the control groups (*P* < 0.05). Either a specific inhibitor of the p38 MAPK pathway or a specific inhibitor of the JNK pathway increased the GRα/GRβ ratio and the Glucocorticoid affinity. None of the specific inhibitors of ERK, PI3K or PKC had any influence on the expression of GR isoforms.

**Conclusion:**

Our results demonstrated that the imbalanced expression of GR isoforms in nasal polyp tissue induced by IL-1β in vitro is mediated through the p38 MAPK and JNK signal pathways.

## Background

Chronic rhinosinusitis (CRS) is a chronic inflammatory disease that involves the mucosa of the nasal cavity and sinus and has a 2-15% incidence rate worldwide [1,2]. Glucocorticoid (GC) therapy is widely used to control mucosal inflammation in CRS cases and to slow the growth of nasal polyps [3-5]. Although GC treatment is effective in the majority of CRS cases, there are still some patients who fail to respond to GC treatment, even at high doses or with supplementary therapies. This phenomenon is termed GC resistance.

The biological action of GC is mediated through the activation of intracellular glucocorticoid receptors (GR). There are two human GR isoforms (GRα and GRβ), and GC’s physiological actions are mainly mediated by GRα, which is far more abundant than GRβ in nasal mucosa [6,7]. GRβ negatively regulates GRα by forming GRα/GRβ heterodimers, reducing the anti-inflammatory effect of GC [8,9]. Recent studies have clearly demonstrated that a higher expression of GRβ led to a lower GRα/GRβ ratio in the nasal polyps of GC-resistant CRS cases than in GC-sensitive nasal polyps, indicating that, to a certain extent, the imbalanced expression of the GR isoforms determines the anti-inflammatory effect of GC therapy and is one of the important factors contributing to GC resistance in CRS [10-12]. However, the detailed mechanism responsible for the GR isoform expression imbalance in the mucosa of GC-resistant CRS cases has not been clearly defined, and merits further investigation.

Recent studies [13-15] have suggested that the abnormal expression of GR isoforms may be related to certain key enzymes in the mitogen activated protein kinase (MAPK) family. However, there has been no discussion about the exact MAPK family signal pathways that may regulate the GR expression in the mucosa of GC-resistant CRS, altering the GRα/GRβ ratio and thereby changing the GC sensitivity of the inflamed nasal mucosa. This study has been designed to explore the upstream signal transduction mechanisms responsible for lowering the GRα/GRβ ratio. In this study, a GC resistance model of nasal polyps induced by interleukin-1 beta (IL-1β) in vitro was developed to measure the relationship between the expression of GR isoforms and p38 MAPK and JNK signal transduction pathways.

## Methods

### Reagents

Primary monoclonal antibodies against GRα, GRβ, phospho-p38MAPK, phospho-JNK and β-actin, as well as a horseradish peroxidase-labeled secondary goat anti-mouse IgG antibody, were purchased from Santa Cruz Biotechnology (USA). IL-1β was purchased from PeproTech (England) and RPMI1640 culture medium was purchased from GIBCO (USA). The specific inhibitors of the p38 MAPK pathway (SB203580), ERK pathway (PD98059), PI3K pathway (LY294002), PKC pathway (Ro31-8220) and JNK pathway (SP600125) were all purchased from Calbiochem (USA). [^3^H]-Dexamethasone was obtained from Amersham (USA). Dexamethasone was purchased from Sigma (USA). The primers and Taqman probes targeting GRα, GRβ, p38 MAPK and JNK were designed and synthesized by Applied Biosystems (USA). All other chemicals used in the study were of analytical grade and obtained from commercial sources.

### Ethics statement

This study received approval from the ethics committee of the Capital Medical University. All patients signed an informed consent form that had been preapproved by the ethics committee.

### Sample collection

Nasal polyp tissues were taken from 15 CRS with nasal polyp patients (6 male, 9 female, aged 41 ± 5 years) who had been admitted to our hospital between March 2011 and June 2011. The patients were each found to have had CRS with nasal polyps for between 8 and 22 months and had received conservative treatment (topical steroid and nasal douching) for 12 weeks based on the standard criteria for CRS with nasal polyps issued in the European Position Paper on Rhinosinusitis and Nasal Polyps 2007 guidelines [16]. On the basis of the immunohistochemical findings, all of the cases were defined as having eosinophil-high inflammation, which, according to the method described by Jankowski [17], was sensitive to GC. Our specimens were obtained during endoscopic surgeries. At the time the specimens were obtained, none of the patients was affected by, nor had they in the last six months been affected by any of the following problems: nasal polyposis, allergic rhinitis, asthma, kidney disease, rheumatoid arthritis, myasthenia gravis, or any infectious diseases; they had not undergone sinus surgery over the last six months; and none of the patients had any history of smoking. All patients suspended the use of angiotensin-converting enzyme inhibitors, beta-agonists and glucocorticoids in the two months prior to their surgery.

### Tissue culturing and intervention

Nasal polyp tissue was cultured according to the reported method [18]. Briefly, all tissue samples were rinsed 5 or 6 times with stroke-physiological saline solution containing penicillin and streptomycin, and each nasal polyp tissue sample was divided into several tissue blocks (approximately 4 mm × 4 mm in size) containing mucous layers. The tissue blocks were then transferred to 6-well culture plates with 1.5 ml RPMI1640 medium (supplemented with 10% fetal bovine serum) and were cultured at 37°C in an atmosphere of 5% CO_2_ for 24 h. The cultured tissue blocks were taken out and divided into several different experiment groups. Each group of samples was exposed to different concentrations of IL-1β with or without the presence of specific inhibitors of MAPK family signal pathways. Two replicate samples from each group were harvested and preserved immediately. One of the two samples was used for protein concentration measurement. The nasal polyp tissue was treated with lysis buffer (150 mM NaCl, 1% Triton X-100, 0.5% deoxycholate, 0.1% SDS, and 50 mM Tris-HCl, pH 7.5) supplemented with a complete protease inhibitor cocktail (Roche Diagnostics; USA) and preserved for Western blot analysis (described below). Protein concentration was measured by the Bradford method. The second sample was used for RNA measurement. Total RNA from the second sample was isolated and preserved at -80°C for the fluorescent quantitative reverse transcription polymerase chain reaction (FQ-RT-PCR) assay described below. For the control group, all steps were the same with the only exception being that all nasal polyp tissues were incubated with medium alone for the same length of time.

### Western blot

An equal amount of denatured proteins (50 μg) was separated in a 12% SDS-polyacrylamide gel (90 V, 1.5 h) and transferred to a nitrocellulose membrane (Millipore; USA) (90 V, 1.5 h). This membrane was then blocked for 2 h at room temperature in 5% (w/v) non-fat dried milk dissolved in a Tris-buffered saline [10 mM Tris (pH 8.0) and 150 mM NaCl] solution containing 0.05% Tween-20. Following this, the membrane was incubated for 1.5 h at room temperature with the indicated primary antibodies (1:500). The membrane was then incubated with the corresponding horseradish peroxidase-labeled secondary goat anti-mouse IgG antibodies. Immunoreactive proteins were measured with the Enhanced Chemiluminescence (ECL) Western blot detection system (Millipore; USA). The β-actin protein served as an endogenous loading control.

### FQ-RT-PCR

Total RNA was reversely transcribed to cDNA using oligo (dT) 18 primers, and PCR amplifications of GRα, GRβ, p38 MAPK and JNK were performed with TaqMan universal master mix. The end products were quantified with an ABI 7900 sequence detector. Each reaction contained 25 μl 2 × Taqman PCR master, 1 μl upstream primer (10 μM), 1 μl downstream primer (10 μM), 1 μl TaqMan probe (10 μM), 5 μl cDNA and 17 μl ddH_2_O, with a total solution volume of 50 μl. The thermocycler parameters were set as: 95°C for 10 min, followed by 40 cycles of 95°C for 30 s and 60°C for 45 s. All reactions were carried out in triplicate. The relative quantity of mRNA was determined using the comparative cycle threshold method, and values were normalized using β-actin as an endogenous control. The primers and probes employed were as follows: GRα, 5′-TGA AAA TGG GTT GGT GCTT CTA-3′ (upstream), 5′-GAC AAG AAT ACT GGA GAT TTG AGT CAA-3′ (downstream), 5′-FAM-CCT GAT GGC ACT TAG CTA TCA GAA GAC CAC AA-TAMRA-3′ (probe); GRβ, 5′-TGG CCA CCC CAA AAG GA-3′ (upstream), 5′-GAG CTC ATC CCA TGC TAA TTA TCC-3′ (downstream), 5′-FAM-AAC TAA CAT GAT TTG TGT CTA TGA AGT GC–TAMR A -3′ (probe); p38MAPK, 5′-GGC TCT GGC GCC TATG G-3′ (upstream), 5′-CCA CAC GTA ACC CCG TTT TT-3′ (downstream), 5′-FAM-TCT GTG TGT GCT GCT TTT GAC A-TAMRA-3′ (probe); JNK, 5′-TAC AGA GCA CCC GAG GTC ATC-3′ (upstream), 5′-AGA GGA TTT TGT GGC AAA CCA-3′ (downstream), 5′-FAM-TGG CAT GGG CTA CAA GGA AAA CG-TAMRA-3′ (probe); β-actin, 5′-TCC CTG GAG AAG AGC TAC GAG-3′ (upstream), 5′-GCC GTG ATC TCC TTC TGC A-3′ (downstream), 5′-FAM- CGC TCT TCC AGC CCT CCT TCC TGG-TAMRA-3′ (probe).

### GC binding assay

[^3^H]dexamethasone binding assay has been used in several previous studies [19,20] to determine GC affinity. This assay was carried out in this research as previously reported. In brief, the polyp tissue blocks were homogenated 3 times and the resulted nasal polyp homogenate was centrifugated (12000 r/min) for 20 min. The homogenate was then isolated and washed 3 times to remove endogenous cortisol before being exposed to [^3^H]dexamethasone ranging between different concentrations (the range 1–50 nM), with or without a 1,000-fold excess unlabelled dexamethasone. Radioactivity was measured in liquid scintillation vials. Specific binding was calculated by subtracting nonspecific binding from the total binding. The equilibrium dissociation constant (Kd) was calculated using SCATCHARD method (by the slope of the curve in the Scatchard plot).

### Statistical analysis

Statistical analysis was carried out using the SPSS Version 16.0 software package. Data sets for a given construct were pooled and averaged. The statistical significance of the difference between control and experimental data sets was determined by analysis of variance (ANOVA) with the least significant difference (LSD) test. Differences with *P*-values less than 0.05 were considered to be statistically significant.

## Results

### Establishment of a GC resistance model of nasal polyps induced by IL-1β in vitro

IL-1β induced a decrease in the GRα/GRβ ratio in a dose-dependent manner in cultured nasal polyps in vitro.

**Figure 1:**
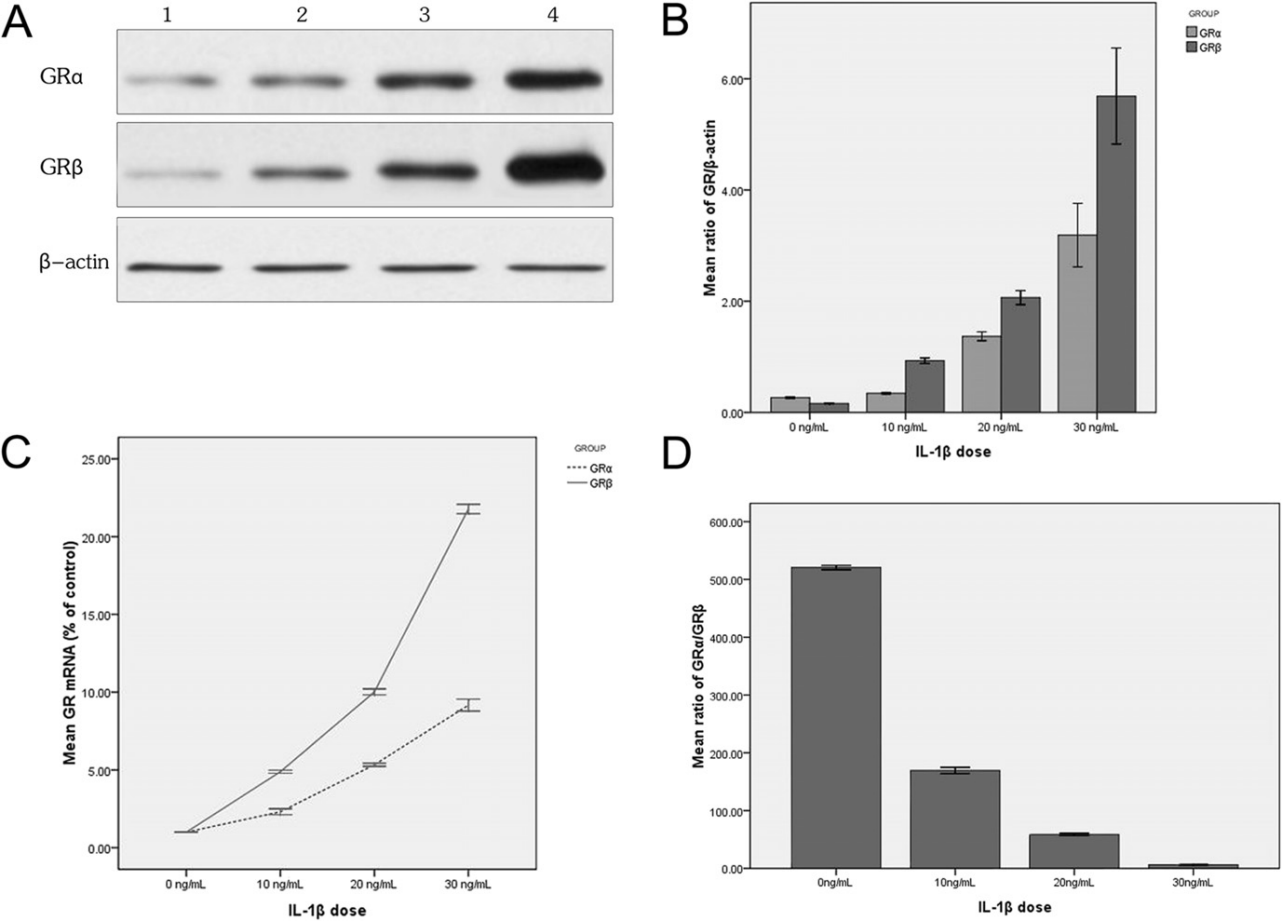
**The expression of GR isoforms induced by IL-1β of different doses in vitro. A** represents Western blot results of IL-1β-induced protein expression of GR isoforms. Lanes 1- 4 represent the GR expression in 0 ng/mL, 10 ng/mL, 20 ng/mL and 30 ng/mL IL-1β-treated groups respectively. The GRα and GRβ expression increased with higher IL-1β dosage. **B** represents the densitometry value (Western blot bands in 1A) ratio of GR isoforms and β-actin. The protein expression of GRα and GRβ increased with the increase of IL-1β dosage, and the GRβ expression increased more significantly than the GRα expression. **C** represents the mRNA ratio of GR isoforms in IL-1β-induced group and the control group (0 ng/mL). The mRNA expression of GRα and GRβ increased with the increase of IL-1β dosage, and the GRβ expression increased more significantly than the GRα expression. **D** represents the GRα/GRβ mRNA expression (measured by FQ-RT-PCR) ratio induced by IL-1β. The GRα/GRβ ratio decreased with the increase of IL-1β dosage in nasal polyp tissue. The data presented above are the means (±SD) of three independent experiments with similar trend.

After having been cultured in the presence of 0 ng/mL, 10 ng/mL, 20 ng/mL and 30 ng/mL IL-1β for 20 h respectively, samples were harvested and GRα and GRβ protein expressions were measured. Low expression of GRα (94 kDa) and GRβ (90 kDa) was detected by using Western blot in the cultured nasal polyp tissue of the control group (0 ng/mL IL-1β), and it showed that the expression level of GRβ was lower than that of GRα (Figure 1A and B). The mRNA and protein expression of GRα and GRβ in the IL-1β-induced nasal polyp tissues increased in a concentration-dependent manner (depending on IL-1β concentration), and the expression of GRβ increased more significantly than that of GRα (Figure 1A,B and C). The GRα/GRβ ratio decreased in a concentration-dependent manner in nasal polyp tissue induced by IL-1β (Figure 1D). The GRα/GRβ ratio of each treatment group at the mRNA and protein levels was significantly lower than that of the control group (*X*^*2*^ = 36.031, *P* = 0.001; *X*^*2*^ = 27.533, *P* = 0.001).

IL-1β induced a decrease in the GRα/GRβ ratio in a time-dependent manner in cultured nasal polyps in vitro.

**Figure 2:**
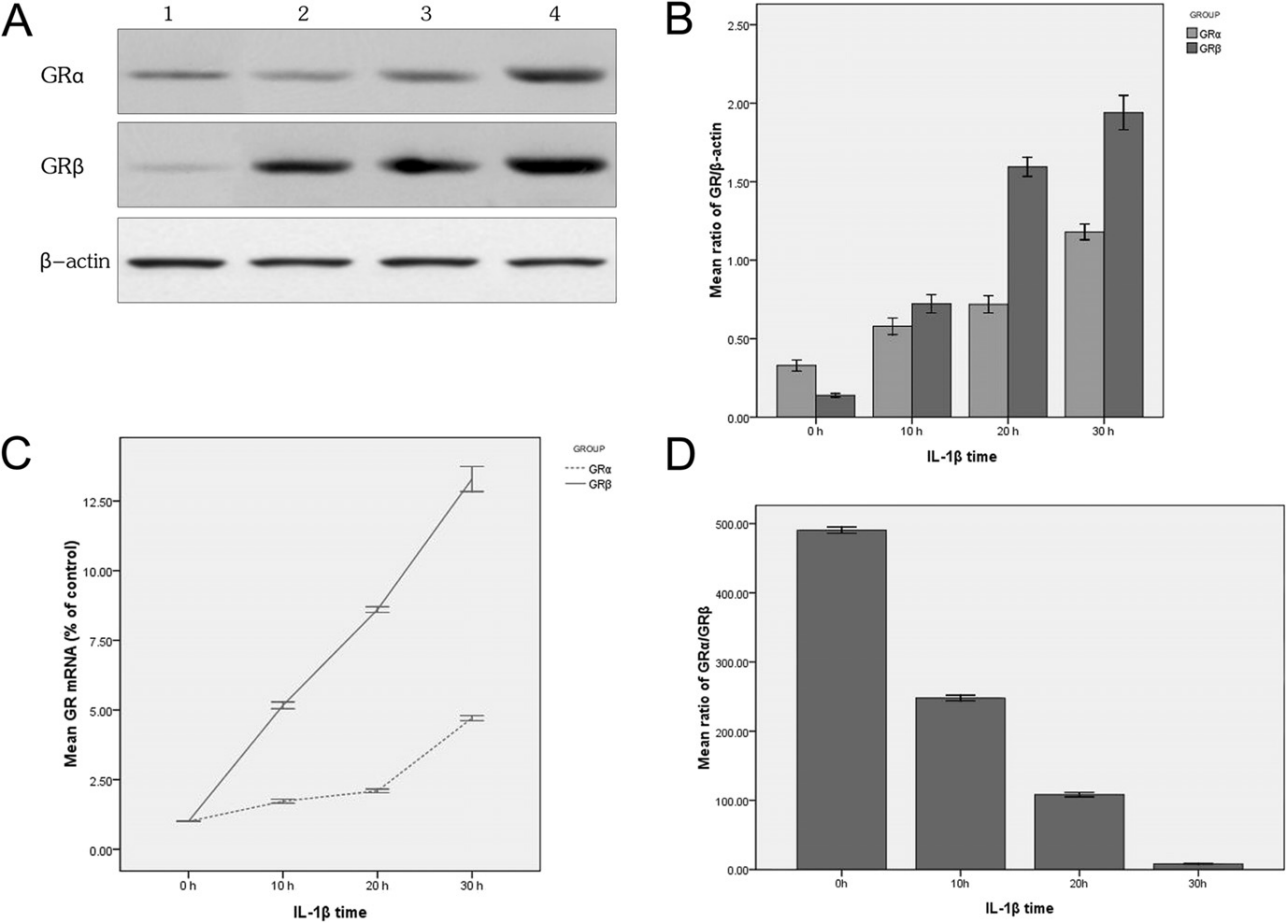
**The expression of GR isoforms induced by IL-1β with different time length in vitro. A** represents Western blot results of IL-1β-induced protein expression of GR isoforms. Lanes 1-4 represent the expression in sample groups treated with IL-1β-treated for 0 h, 10 h, 20 h and 30 h respectively. The GRα and GRβ expression increased with longer IL-1β-inducing time. **B** represents the densitometry value (Western blot bands in 2A) ratio of GR isoforms and β-actin. The protein expression of GRα and GRβ increased as the IL-1β-inducing time was increased, and the GRβ expression increased more significantly than the GRα expression. **C** represents the ratio of GR isoforms mRNA between IL-1β-induced groups and the control group (0 h). The mRNA expression of GRα and GRβ increased as the IL-1β-inducing time was increased, and the GRβ expression increased more significantly than the GRα expression. **D** represents the GRα/GRβ mRNA expression (measured by FQ-RT-PCR) ratio induced by IL-1β. The GRα/GRβ ratio decreased with the increase of IL-1β-inducing time in nasal polyp tissue. The data presented are means ± SD of three independent experiments with similar trend.

Nasal polyp tissues were cultured in the presence of 20 ng/mL IL-1β for 0 h, 10 h, 20 h and 30 h respectively. IL-1β induced the expression of GRα and GRβ in a time-dependent manner, and the expression of GRβ increased more significantly than that of GRα (Figure 2A,B and C). IL-1β treatment led to lower GRα/GRβ ratio in a time-dependent manner (Figure 2D). The GRα/GRβ ratio at the mRNA and protein levels observed in each treatment group was significantly lower than that of the control group (*X*^*2*^ = 52.321, *P* = 0.002; *X*^*2*^ = 38.742, *P* = 0.001).

The same set of IL-1β concentrations also induced an activation of p38 MAPK and JNK signal pathways in a similar dose-dependent manner.

**Figure 3:**
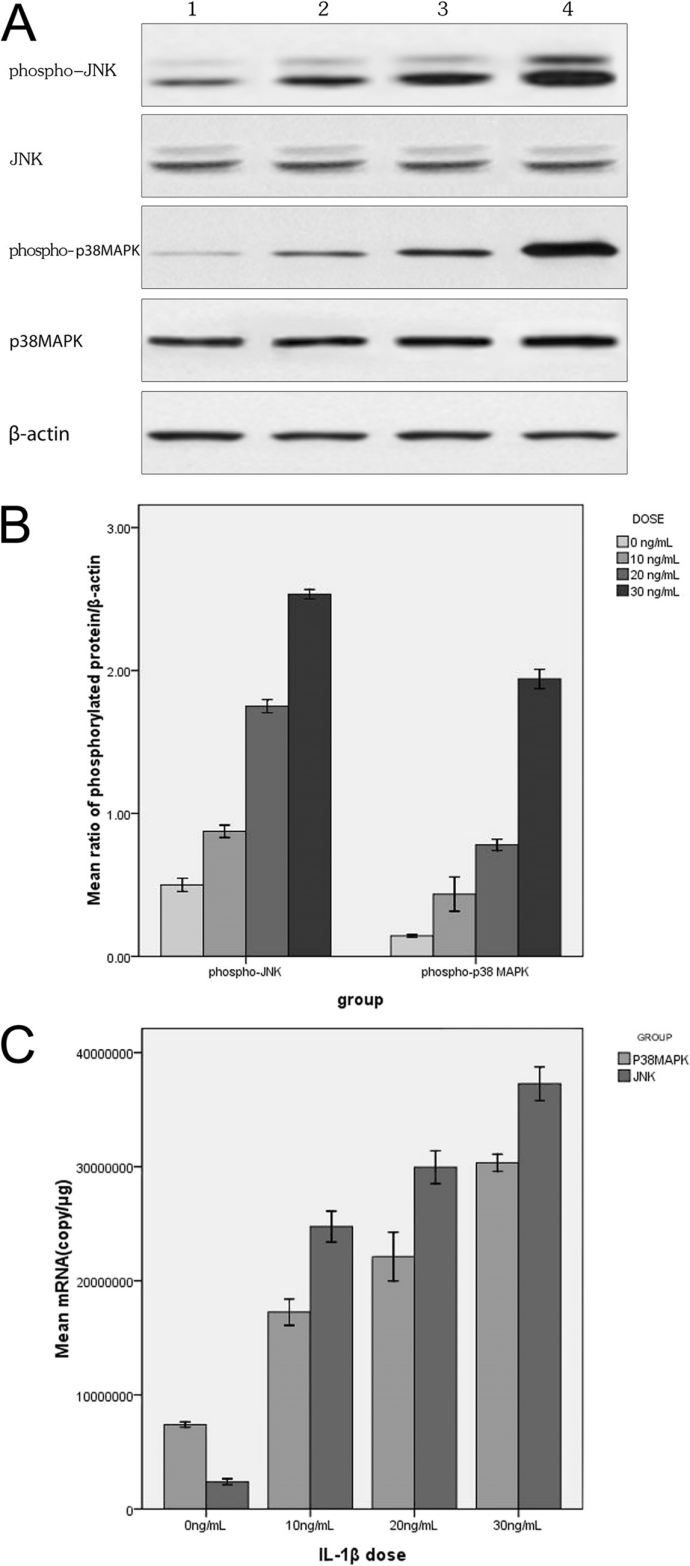
**P38 MAPK and JNK expression induced by IL-1β of different doses in vitro. A** represents Western blot results of IL-1β-induced protein expression. Lanes 1-4 represent the expression in the groups treated with IL-1β 0 ng/mL, 10 ng/mL, 20 ng/mL and 30 ng/mL respectively. The expression of phospho-p38 MAPK and phospho-JNK increased as the IL-1β dosage was increased. **B** represents the densitometry value (Western blot bands in 3A) ratio of phospho-p38 MAPK, phospho-JNK and β-actin. The expression of phospho-p38 MAPK and phospho-JNK increased as the IL-1β dosage was increased. **C** represents FQ-RT-PCR results of IL-1β-induced mRNA expressions of p38 MAPK and JNK with similar trends as protein expressions. The data presented are means (±SD) of three independent experiments with similar trend.

0 ng/mL, 10 ng/mL, 20 ng/mL and 30 ng/mL of IL-1β were used to induce nasal polyp tissues for 20 h in vitro respectively. Both phospho-p38 MAPK (39 kDa) and phospho-JNK (46 kDa/54 kDa) protein and mRNA were extracted for quantitative measurements. A low expression of phospho-p38 MAPK and phospho-JNK was observed in the control group. IL-1β led to higher phospho-p38 MAPK and phospho-JNK expression at the protein (Figure 3A and B) and mRNA (Figure 3C) levels in a concentration-dependent manner in nasal polyp tissues in vitro. The mRNA and protein expression of p38 MAPK and JNK in each IL-1β-induced group was significantly higher than that in the control group (*X*^*2*^ = 18.071, *P* = 0.001; *X*^*2*^ = 17.371, *P* = 0.002; *X*^*2*^ = 21.913, *P* = 0.001; *X*^*2*^ = 33.652, *P* = 0.001).

It was found that p38 MAPK and JNK pathways but not PI3K, ERK or PKC were responsible for the regulation of the GRα/GRβ ratio and GC affinity in IL-1β-induced polyps.

**Figure 4:**
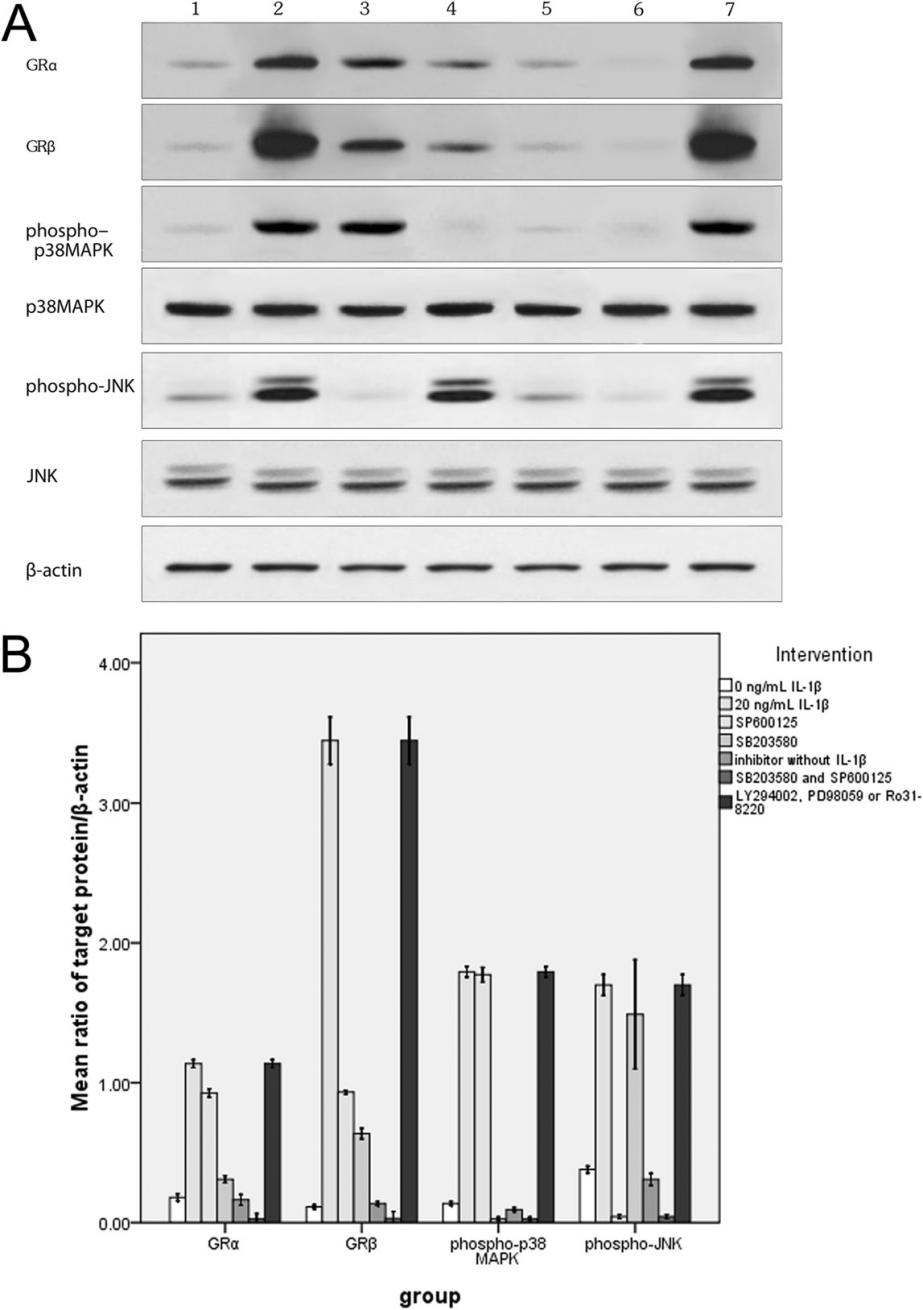
**Involvement of p38 MAPK and JNK pathways in the regulation of GR isoform expression. A**: Lane 1: Trace expressions of all target proteins in the control group; Lane 2: Increased expressions of all target proteins in the 20 ng/ml IL-1β group; Lane 3: JNK-specific inhibitor (SP600125) decreased the expressions of GR isoforms and phospho-JNK, but not that of phospho-p 38MAPK; Lane 4: P 38MAPK-specific inhibitor (SB203580) decreased the expressions of GR isoforms and phospho-p 38MAPK, but not that of phospho-JNK; Lane 5: Trace expressions of all target proteins were detected in nasal polyp tissue incubated with SB203580 or SP600125 but without IL-1β induction. Lane 6: SB203580 and SP600125 inhibited almost all expressions of GR isoforms, phospho-p 38MAPK and phospho-JNK in IL-1β-induced nasal polyp in vitro. Lane 7: Specific inhibitors (LY294002, PD98059 or Ro31-8220) of the PI3K, ERK or PKC pathways did not influence the expressions of GR isoforms, phospho-p 38MAPK and phospho-JNK in IL-1β-induced nasal polyp in vitro. **B** represents the densitometry value (Western blot bands in 4A) ratio of above-mentioned target proteins and β-actin. The protein levels shown are representative of three independent experiments with similar trend. (Western blot).

Specific inhibitors (SB203580 and SP600125) of the p38 MAPK or JNK signal pathways were separately added (at a dose of 5 μM) to the culture medium 30 min before the induction with 20 ng/mL IL-1β. The expressions of GR isoforms, phospho-p38 MAPK and phospho-JNK were measured 20 h after IL-1β induction (Figure 4). The results showed that 5 μM SB203580 (a specific inhibitor of p38 MAPK) significantly lowered the expression of phospho-p38 MAPK, while the expression of phospho-JNK did not differ from that in the control group (0 μM SB203580). Meanwhile, the expressions of GRα and GRβ also decreased, with GRβ expression having decreased more significantly leading to a higher GRα/GRβ ratio. The GRα/GRβ ratio in the SB203580-treated group was significantly higher than that of the control group (*X*^*2*^ = 17.431, *P* = 0.001). Treatment with 5 μM SP600125 (specific inhibitor of JNK) lead to significantly lower expression of phospho-JNK, while the expression of phospho-p38 MAPK was not found to be significantly different from that in the control group (0 μM SP600125). It was found that the expressions of GRα and GRβ also decreased, with the GRα/GRβ ratio of the 5 μM SP600125 group significantly higher than that of the control group (*X*^*2*^ = 18.071, *P* = 0.001). Further, SB203580 exhibited a much stronger inhibitive effect on GRα and GRβ expressions than SP600125 (Figure 4A and B). After both SB203580 and SP600125 were added simultaneously to the medium with IL-1β induction, almost all expressions of GRα and GRβ were inhibited (Figure 4A and B). Moreover, incubation with 5 μM SB203580 or 5 μMSP600125 without IL-1β induction did not reveal in changes the GR isoform expressions at all. Specific inhibitors (LY294002, PD98059 or Ro31-8220) of the PI3K, ERK or PKC signal pathways were added separately (at a dose of 20 μM) to the culture medium 30 min before they were induced with 20 ng/mL IL-1β. The expressions of GR isoforms in LY294002, PD98059 or Ro31-8220 treated groups were not found to be significantly different from that in the 20 ng/mL IL-1β-induced group (Figure 4A and B). The trend of GRα and GRβ mRNAs demonstrated the same patterns as that of those proteins in the above experiments (data not shown). In addition, GC affinity and sensitivity were determined by GC binding assay. GC affinity for 20 ng/mL IL-1β group (Kd = 20.89 ± 1.54) dropped more significantly than that for the control group (Kd = 4.45 ± 0.54, *P* < 0.01). Our results also showed that the GC affinity and sensitivity increased after the nasal polyp was incubated with SB203580 (Kd = 8.05 ± 1.36, *P* < 0.01) or SP600125 (Kd = 12.40 ± 1.83, *P* < 0.01), but did not change when incubated with LY294002, PD98059 or Ro31-8220 (Kd = 20.76 ± 1.89, *P* > 0.05).

## Discussion

A decrease in the GRα/GRβ ratio in nasal polyps is one of the important features of GC-resistant CRS. However, its etiology has remained unexplained, and there is currently no direct and effective treatment for GC-resistant CRS. In this study, IL-1β was used to induce cultured nasal polyp tissues in vitro. With the IL-1β induction, the GRα/GRβ ratio declined in both a time-dependent manner and a concentration-dependent manner, which demonstrates the successful establishment of a GRα/GRβ imbalance model in vitro. We also found that after cultured nasal polyps were induced with the same set of IL-1β concentrations, the p38 MAPK and JNK signal pathways were also activated, as the expression of key enzymes of both pathways increased in a similar concentration-dependent manner. Furthermore, the intervention with specific inhibitors of the p38 MAPK and JNK pathways resulted in an increased GRα/GRβ ratio and a recovery of GC affinity in IL-1β-induced polyps. These results suggest that the p38 MAPK and JNK signal pathways may play an important role in the IL-1β-induced GRα/GRβ imbalance in nasal polyps and may be related to GC resistance in nasal polyps. Our results also suggest that blocking these signal pathways may lead to a recovery of GC sensitivity in GC resistance nasal polyps.

IL-1β is one of the important cytokines involved in the etiology of nasal mucosal inflammation. Histological studies have revealed that its expression increased in the mucosa and sinus secretions of rhinosinusitis patients, and this cytokine was confirmed to be associated with rhinosinusitis [21,22]. In our own study, we found that IL-1β could induce a reduction of the GRα/GRβ ratio in dose- and time-dependent manners in cultured nasal polyps in vitro. Further, the expression of GRβ increased more than GRα at the mRNA and protein levels, suggesting that the increase in GRβ synthesis is the main cause of the decreased GRα/GRβ ratio induced by IL-1β in vitro. Our results are consistent with other reported results based on GC resistance models involving human pulmonary epithelial cells, spleen cell and intestinal epithelial cell lines induced by IL-1β in vitro [23-25].

Although recent studies have confirmed that the decreased GRα/GRβ ratio is one of the important features in GC-resistant CRS, the signal transduction mechanism responsible has not yet been fully identified, meaning that further research is needed. Before now, it has been suggested that p38 MAPK and JNK are involved in the signal transduction of GR. This study examined the activation of the p38 MAPK and JNK signal pathways in the IL-1β-induced samples, and found that the expression of activated key enzymes (phospho-p38 MAPK or phospho-JNK) increased in the same manner as the GRβ/GRα ratio. The above results suggest that the p38 MAPK and JNK signal pathways may be associated with the change of GRα/GRβ ratio. Furthermore, the ratio of GRα/GRβ and GC affinity were observed to increase after the p38 MAPK or JNK pathways were blocked by specific inhibitors, SB203580 and SP600125, respectively, indicating that the p38 MAPK and JNK signal pathways are indeed involved in regulating the ratio of GR isoforms and GC sensitivity in IL-1β-induced nasal polyp tissues in vitro. In order to further exclude the possibility of SB203580 or SP600125 directly affecting GR isoform expression, we performed a negative control experiment (samples not induced with IL-1β). Neither SB203580 nor SP600125 led to changs in GR isoform expression in this negative control experiment. The above results show that the synthesis of GR isoforms may be regulated through p38 MAPK and JNK signal pathways in the IL-1β induced nasal polyps. We believe that the change in the GRα/GRβ ratio may cause the insensitivity of nasal polyps to GC. Blocking the above signal pathways helped to increase the GRα/GRβ ratio, which may improve GC sensitivity in nasal polyps. Our results are consistent with recent studies reported by other researchers on the signal pathways involved with GR expression and GC sensitivity in brain tissue [26].

Although our study showed that both the p38 MAPK and JNK signal pathways were involved in the regulation of GR isoform expression in IL-1β-induced nasal polyps in vitro, more research needs to be carried out in order to determine how these two pathways regulate this process. In this study, we found that both SB203580 and SP600125 can influence the expression of GR isoforms, but blocking the p38 MAPK signal pathway with SB203580 cannot change the expression of a key enzyme in the JNK pathway, and vice versa. The above evidence indicates that the two signal pathways involved in regulating GR expression operate in parallel, and there was no significant interaction observed. Our study also found that SB203580 has a more significant effect on GR isoform expression and GC affinity than SP600125, suggesting that the p38 MAPK may be a more important pathway than the JNK pathway.

In contrast to the results of our own experiments described above, a recent study by Zijlstra and colleagues [27] reported that GR expression and GC sensitivity in IL-17-induced bronchial epithelial cells (16HBE) were regulated through the phosphoinositide-3-kinase (PI3K) pathway, and not through the p38 MAPK and ERK signal pathways. Another study by Miller et al. [28] on GC sensitivity in clones of the human acute lymphoblastic CEM cell line confirmed that the JNK, p38 MAPK, ERK and PKC signal pathways were all involved in GR signal transduction in CEM cells, with JNK and ERK being the main upstream pathways and the p38 MAPK and PKC pathways playing a minor role. To further validate the involvement of PI3K, ERK and PKC signal transduction pathways in regulating GR expression and GC resistance in IL-1β-induced nasal polyps in vitro, the specific inhibitors of these pathways were used to block signal pathways in this study. No noticeable changes of GR isoforms and GC affinity were observed after PI3K, ERK or PKC pathways were blocked, which suggests that these pathways were not involved in the regulation of GR expression and GC sensitivity in the IL-1β-induced nasal polyps in vitro. Based on the aforementioned studies and our own present investigation, we believe that these results on one hand demonstrate the complexity of the signal transduction mechanisms involved in regulating the GR isoforms, and on the other, highlight that the upstream signal pathways of GR and their network have obvious tissue specificity and inducer specificity. Different tissues, induced by different cytokines, may regulate GR isoform expression via different signal transduction pathways. Therefore, we propose that only by further investigating the specific upstream signal transduction pathways involved in GR regulation in a CRS mucosal inflammation environment will it be possible to improve the treatment effect of GC resistance in CRS by targeting its specific signal pathways.

## Conclusions

In summary, our study showed that the p38 MAPK and JNK signal pathways are involved in regulating the expression of GR isoforms in IL-1β-induced nasal polyps in vitro, as their activation resulted in a decrease of GRα/GRβ ratio. Further, our results suggest that these two pathways are involved in the etiology of GC-resistant CRS. Our findings also provide deeper insights into the pathogenesis of GC resistance in CRS and provide further clues as to which pathways should be targeted in developing a more precise treatment for GC-resistant CRS. However, the precise upstream signal mechanisms involved in GR regulation are very complex. In this study, only five signal pathways were investigated. A complete elucidation of the pathological mechanisms of GC-resistant CRS requires further thorough study on all other related pathways.

## Abbreviations

GR: Glucocorticoid receptor
GC: Glucocorticoid
CRS: Chronic rhinosinusitis
MAPK: Mitogen activated protein kinase
IL-1β: Interleukin-1 beta
